# Supplementary material for: EnzML: multi-label prediction of enzyme classes using InterPro signatures
Source: BMC Bioinformatics. 2012 Apr 25;13:61. doi: 10.1186/1471-2105-13-61 (PMC3483700; doi:10.1186/1471-2105-13-61)
Supplement: Addtional file 5 — The Java code to format the data files, evaluate and predict. The file enzml_java_code.tar.gz contains the Java code used to format database data to ARFF and XML formats, to execute cross and train-test (jackknife) evaluations and to record evaluation results to database. More information is included in the readme.txt file and the Javadoc files. The code can be used with a MySQL database. To use a different database software, other JDBC drivers might be required. [file 1471-2105-13-61-S5.gz › java_code/utils/doc/cern/jet/random/engine/MersenneTwister.html]

MersenneTwister


---


|  |  |  |
| --- | --- | --- |
| **Overview** \| **Package** \| **Class** \| **Use** \| **Tree** \| **Deprecated** \| **Index** \| **Help** | |  |
| PREV CLASS   **NEXT CLASS** | **FRAMES**    **NO FRAMES**     **All Classes** |  |
| SUMMARY: NESTED \| FIELD \| CONSTR \| METHOD | DETAIL: FIELD \| CONSTR \| METHOD |  |


---


## cern.jet.random.engine Class MersenneTwister

```
java.lang.Object
  cern.colt.PersistentObject
      edu.cornell.lassp.houle.RngPack.RandomElement
          edu.cornell.lassp.houle.RngPack.RandomSeedable
              cern.jet.random.engine.RandomEngine
                  cern.jet.random.engine.MersenneTwister
```

**All Implemented Interfaces:**: DoubleFunction, IntFunction, java.io.Serializable, java.lang.Cloneable

---

``` public class MersenneTwister extends RandomEngine ```

MersenneTwister (MT19937) is one of the strongest uniform pseudo-random
number generators known so far; at the same time it is quick. Produces
uniformly distributed int's and long's in the closed
intervals [Integer.MIN\_VALUE,Integer.MAX\_VALUE] and
[Long.MIN\_VALUE,Long.MAX\_VALUE], respectively, as well as
float's and double's in the open unit intervals
(0.0f,1.0f) and (0.0,1.0), respectively. The seed can be
any 32-bit integer except 0. Shawn J. Cokus commented that perhaps
the seed should preferably be odd.

**Quality:** MersenneTwister is designed to pass the k-distribution test.
It has an astronomically large period of 2^19937^-1
(=10^6001^) and 623-dimensional equidistribution up to 32-bit
accuracy. It passes many stringent statistical tests, including the diehard test of G. Marsaglia
and the load test of P. Hellekalek and S. Wegenkittl.

**Performance:** Its speed is comparable to other modern generators (in
particular, as fast as java.util.Random.nextFloat()). 2.5 million
calls to raw() per second (Pentium Pro 200 Mhz, JDK 1.2, NT). Be
aware, however, that there is a non-negligible amount of overhead required to
initialize the data structures used by a MersenneTwister. Code like

```
 double sum = 0.0;
 for (int i = 0; i < 100000; ++i) {
        RandomElement twister = new MersenneTwister(new java.util.Date());
        sum += twister.raw();
 }
```

will be wildly inefficient. Consider using

```
 double sum = 0.0;
 RandomElement twister = new MersenneTwister(new java.util.Date());
 for (int i = 0; i < 100000; ++i) {
        sum += twister.raw();
 }
```

instead. This allows the cost of constructing the MersenneTwister object to
be borne only once, rather than once for each iteration in the loop.

**Implementation:** After M. Matsumoto and T. Nishimura,"Mersenne Twister: A 623-Dimensionally Equidistributed Uniform Pseudo-Random Number Generator"
, ACM Transactions on Modeling and Computer Simulation, Vol. 8, No. 1,
January 1998, pp 3--30.

More info on Masumoto's homepage. More info on Pseudo-random number generators is on the Web. Yet some more info. The correctness of this implementation has been verified against the published output sequence mt19937-2.out of the C-implementation mt19937-2.c. (Call test(1000) to print the sequence). Note that this implementation is **not synchronized**. **Details:** MersenneTwister is designed with consideration of the flaws of various existing generators in mind. It is an improved version of TT800, a very successful generator. MersenneTwister is based on linear recurrences modulo 2. Such generators are very fast, have extremely long periods, and appear quite robust. MersenneTwister produces 32-bit numbers, and every k-dimensional vector of such numbers appears the same number of times as k successive values over the period length, for each k <= 623 (except for the zero vector, which appears one time less). If one looks at only the first n <= 16 bits of each number, then the property holds for even larger k, as shown in the following table (taken from the publication cited above): | | | | | | | | | | | | | | | | --- | --- | --- | --- | --- | --- | --- | --- | --- | --- | --- | --- | --- | --- | | n | 1 | 2 | 3 | 4 | 5 | 6 | 7 | 8 | 9 | 10 | 11 | 12 .. 16 | 17 .. 32 | | k | 19937 | 9968 | 6240 | 4984 | 3738 | 3115 | 2493 | 2492 | 1869 | 1869 | 1248 | 1246 | 623 | MersenneTwister generates random numbers in batches of 624 numbers at a time, so the caching and pipelining of modern systems is exploited. The generator is implemented to generate the output by using the fastest arithmetic operations only: 32-bit additions and bit operations (no division, no multiplication, no mod). These operations generate sequences of 32 random bits (int's). long's are formed by concatenating two 32 bit int's. float's are formed by dividing the interval [0.0,1.0] into 2^32^ sub intervals, then randomly choosing one subinterval. double's are formed by dividing the interval [0.0,1.0] into 2^64^ sub intervals, then randomly choosing one subinterval. **Version:** : 1.0, 09/24/99 **Author:** : wolfgang.hoschek@cern.ch **See Also:**: `Ranmar`, `Ranlux`, `Ranecu`, `Random`, Serialized Form --- | **Field Summary** | | | --- | --- | | `static int` | `DEFAULT_SEED` | **Constructor Summary** `MersenneTwister()`           Constructs and returns a random number generator with a default seed, which is a **constant**. `MersenneTwister(java.util.Date d)`           Constructs and returns a random number generator seeded with the given date. `MersenneTwister(int seed)`           Constructs and returns a random number generator with the given seed. | **Method Summary** | | | --- | --- | | `java.lang.Object` | `clone()`           Returns a copy of the receiver; the copy will produce identical sequences. | | `int` | `nextInt()`           Returns a 32 bit uniformly distributed random number in the closed interval [Integer.MIN\_VALUE,Integer.MAX\_VALUE] (including Integer.MIN\_VALUE and Integer.MAX\_VALUE). | **Methods inherited from class cern.jet.random.engine.RandomEngine** `apply, apply, makeDefault, nextDouble, nextFloat, nextLong, raw` **Methods inherited from class edu.cornell.lassp.houle.RngPack.RandomSeedable** `ClockSeed, ClockSeed` **Methods inherited from class edu.cornell.lassp.houle.RngPack.RandomElement** `choose, choose, gaussian, gaussian, powlaw, raw, raw, uniform` **Methods inherited from class java.lang.Object** `equals, getClass, hashCode, notify, notifyAll, toString, wait, wait, wait` **Field Detail** ### DEFAULT\_SEED ``` public static final int DEFAULT_SEED ``` **See Also:**: Constant Field Values **Constructor Detail** ### MersenneTwister ``` public MersenneTwister() ``` : Constructs and returns a random number generator with a default seed, which is a **constant**. Thus using this constructor will yield generators that always produce exactly the same sequence. This method is mainly intended to ease testing and debugging. --- ### MersenneTwister ``` public MersenneTwister(java.util.Date d) ``` : Constructs and returns a random number generator seeded with the given date. **Parameters:**: `d` - typically new java.util.Date() --- ### MersenneTwister ``` public MersenneTwister(int seed) ``` : Constructs and returns a random number generator with the given seed. **Parameters:**: `seed` - should not be 0, in such a case MersenneTwister.DEFAULT\_SEED is silently substituted. **Method Detail** ### clone ``` public java.lang.Object clone() ``` : Returns a copy of the receiver; the copy will produce identical sequences. After this call has returned, the copy and the receiver have equal but separate state. : **Overrides:**: `clone` in class `PersistentObject` : **Returns:**: a copy of the receiver. --- ### nextInt ``` public int nextInt() ``` : Returns a 32 bit uniformly distributed random number in the closed interval [Integer.MIN\_VALUE,Integer.MAX\_VALUE] (including Integer.MIN\_VALUE and Integer.MAX\_VALUE). : **Specified by:**: `nextInt` in class `RandomEngine` --- | | | | | --- | --- | --- | | **Overview** \| **Package** \| **Class** \| **Use** \| **Tree** \| **Deprecated** \| **Index** \| **Help** | | | | PREV CLASS   **NEXT CLASS** | **FRAMES**    **NO FRAMES**     **All Classes** | | | SUMMARY: NESTED \| FIELD \| CONSTR \| METHOD | DETAIL: FIELD \| CONSTR \| METHOD | | ---
